# Aucubin Impeded Preosteoclast Fusion and Enhanced CD31^hi^ EMCN^hi^ Vessel Angiogenesis in Ovariectomized Mice

**DOI:** 10.1155/2022/5226771

**Published:** 2022-11-09

**Authors:** Ziyi Li, Chang Liu, Xiaoli Liu, Na Wang, Liu Gao, Xiaoxue Bao, Sijing Liu, Peng Xue

**Affiliations:** ^1^Department of Endocrinology, The Third Hospital of Hebei Medical University, Shijiazhuang 050051, China; ^2^Key Orthopaedic Biomechanics Laboratory of Hebei Province, Shijiazhuang 050051, China; ^3^Department of Pediatric Dentistry, School and Hospital of Stomatology & Hebei Key Laboratory of Stomatology, Hebei Medical University, Shijiazhuang 050017, China; ^4^Editorial Department of Hebei Medical University, Hebei Medical University, Shijiazhuang 050017, China

## Abstract

Osteogenesis is tightly correlated with angiogenesis during the process of bone development, regeneration, and remodeling. In addition to providing nutrients and oxygen for bone tissue, blood vessels around bone tissue also secrete some factors to regulate bone formation. Type H vessels which were regulated by platelet-derived growth factor-BB (PDGF-BB) were confirmed to couple angiogenesis and osteogenesis. Recently, preosteoclasts have been identified as the most important source of PDGF-BB. Therefore, inhibiting osteoclast maturation, improving PDGF-BB secretion, stimulating type H angiogenesis, and subsequently accelerating bone regeneration may be potent treatments for bone loss disease. In the present study, aucubin, an iridoid glycoside extracted from *Aucuba japonica* and *Eucommia ulmoides*, was found to inhibit bone loss in ovariectomized mice. We further confirmed that aucubin could inhibit the fusion of tartrate-resistant acid phosphatase (TRAP)^+^ preosteoclasts into mature osteoclasts and indirectly increasing angiogenesis of type H vessel. The underlying mechanism is the aucubin-induced inhibition of MAPK/NF-*κ*B signaling, which increases the preosteoclast number and subsequently promotes angiogenesis via PDGF-BB. These results prompted that aucubin could be an antiosteoporosis drug candidate, which needs further research.

## 1. Introduction

The functional changes of osteoblasts and osteoclasts cause dynamic changes in the skeletal system. Uncoordinated action between osteoclasts and osteoblasts might result in many bone loss diseases, such as osteoporosis. Recently, our understanding of bone disease has expanded beyond the bone itself, and the relationship between the bone and bone microenvironment, especially blood vessels, has been gradually recognized. Type H vessels, a specific capillary subtype featuring CD31 and endomucin (CD31^hi^Emcn^hi^) markers, can provide nutrients and oxygen for bone tissue [1, 2]. Importantly, type H vessels are further identified around osteoprogenitors and interconnect the processes of bone formation and bone absorption during bone regeneration [3, 4]. The combination of angiocrine factors derived by type H vessels, osteoblasts, and osteoclasts could couple the interreaction of the bone microenvironment as well as the coordinating of angiogenesis and osteogenesis [5–9]. However, the relative abundance of type H vessels is low and reduces with the increasing of aging and the progressing of diseases [2].

A previous study showed that PDGF-BB produced by preosteoclasts had the ability to promote type H vessel angiogenesis and subsequently increase osteogenesis [10]. However, with the maturation of osteoclasts, the amount of PDGF-BB secreted by osteoclasts gradually decreased. Therefore, inhibiting the maturation of preosteoclasts can inhibit bone resorption and importantly promote bone formation through promoting angiogenesis of type H vessels and might be promising therapeutic strategies for osteoporosis.

Aucubin is an iridoid glycoside extracted from *Aucuba japonica* and *Eucommia ulmoides* leaves. It has multiple functions, such as anti-inflammatory, antioxidative, cardioprotective, and neuroprotective effects [11–16]. In recent years, studies have found that aucubin is also related to bone metabolism by increasing bone formation [17, 18]. In addition, aucubin has been reported to promote angiogenesis in a mouse hindlimb ischaemia model [19]. Aucubin can play an antioxidant role by suppressing the NF-*κ*B signaling pathway [11, 20], which is also a very important signal during the process of osteoclastogenesis. However, the effects of aucubin on osteoclasts and type H vessels angiogenesis remain unknown. In this research, we explored whether aucubin could inhibit the maturation of preosteoclasts and amplify angiogenesis by upregulating the secretion of PDGF-BB.

## 2. Materials and Methods

### 2.1. Animals and Treatments

All experiments of animal in this present research were under the supervision of the ethics committee of the Third Hospital of Hebei Medical University. The BALB/c female mice were purchased from the animal experimental center of Hebei Medical University and maintained until use. All mice were bred in a standard environment room with ad libitum access to water and food. All the animals were randomly assigned to the following three groups: (1) the sham group (sham-operated group+PBS), (2) the ovariectomy (OVX+PBS) group, and (3) the aucubin group (OVX+aucubin). Ovariectomy was performed by bilateral removal of ovaries at the 12 weeks old. One week after OVX operation, the aucubin group received 5 mg/kg aucubin by intraperitoneal injection every two days. Correspondingly, the OVX group was injected with the same amount of PBS at the same frequency. All mice were sacrificed one month later; blood and femurs were collected for further experiments.

### 2.2. Micro-CT Analysis

Micro-CT scans in isolated bones were performed using SkyScan1176 (Bruker, Belgium) *μ*CT scanner. 9 *μ*m per pixel resolution was set in the study. The scanning voltage is 65 kV, and the current is 153 *μ*A. The trabecular parameters of femoral metaphysis were analyzed by data analysis software (CTAn, v1.9, SkyScan) and 3D model visualization software (CTVol, v2.0, SkyScan). The 3D analysis of trabecular bone was performed by creating cross-sectional images of femur. The trabecular parameters were measured by trabecular bone volume/total volume (BV/TV, %), trabecular number (Tb.N, mm^−1^), trabecular separation (Tb.Sp, *μ*m), and trabecular thickness (Tb.Th, mm), and the differences among the groups were compared.

### 2.3. Haematoxylin and Eosin (HE) Staining and Tartrate-Resistant Acid Phosphatase (TRAP) Staining

HE staining and TRAP staining were performed on 4 *μ*m sections of the femur after 3 weeks of bone decalcification in 10% EDTA solution. For HE staining, the 4 *μ*m thick section samples were processed for staining after fixation, decalcification and paraffin embedding. The images were obtained by an optical microscope, and the relevant bone histological parameters were calculated. For TRAP staining, a TRAP staining kit (Sigma-Aldrich) was used after the tissue was fixed and embedded according to the instructions. After trap staining, samples were analyzed by optical microscope. TRAP-positive cells with more than three nuclei were defined as osteoclasts, and TRAP-positive cells with less than three nuclei were preosteoclasts.

### 2.4. Immunofluorescent Analyses of Sample Sections

For immunofluorescent (IF) staining, the femur samples were cut into 16 *μ*m thick sections after being fixed for 24 hours, decalcified for 3 weeks, and embedded in 8% gelatin. Briefly, the 16 *μ*m thick sections were first washed with 1% PBST (1% Triton X-100 dissolve in PBS) for 30 min three times before the slices were incubated with 5% BSA at 37°C for 1 h. Then, the samples were incubated with primary antibodies against CD31 (Abcam, MA, USA), EMCN (Abcam, MA, USA) and osteocalcin (Abcam, MA, USA) overnight at 4°C. Finally, the corresponding fluorescence-conjugated secondary antibodies were stained at 37°C for 1 h.

### 2.5. Cell Culture

RAW264.7 cells, the macrophage cell line, was purchased from the Cell Bank of the Chinese Academy of Sciences (Shanghai, China). *α*-MEM supplemented with 10% heat-inactivated foetal bovine serum (FBS) and 1% penicillin-streptomycin was used to culture the RAW264.7 cells. To induce osteoclast differentiation, the cells were treated with 50 ng/mL receptor activator for nuclear factor *κ*B ligand (RANKL) with different concentrations of aucubin (0, 1, or 5 *μ*M) for 6 days. Microvascular endothelial cells of mice (MMECs) were purchased from Procell (Wuhan, China) and cultured in endothelial cell culture medium (ECM). All cells were cultured at 37°C with 5% CO_2_.

### 2.6. Cell Viability Assay

Cell viability was performed by a cell Counting Kit-8 (CCK-8) assay (Zomanbio, Beijing, China) according to the manufacturer's instructions. First, RAW264.7 cells were seeded onto 96-well plates at a density of 5 × 10^3^ cells per well. After treatment with aucubin (0, 1, or 5 *μ*M) for 24, 48, and 72 hours, 10 *μ*L CCK-8 solution was added. Finally, the absorbance at 450 nm was measured by a microplate spectrophotometer (BioTek Instruments, San Jose, CA, USA) after being incubated at 37°C for another 4 h.

### 2.7. TRAP Staining of Cells

The macrophage cell lines were fixed with 4% paraformaldehyde after several days of osteoclastogenesis induction. Then, TRAP staining was performed using a TRAP staining kit (Sigma-Aldrich, St. Louis, USA) according to the manufacturers' instructions. After trap staining, samples were analyzed by optical microscope. Same as tissue samples, TRAP+ mononuclear cells and multinucleated cells with more than three nuclei were identified as preosteoclasts and osteoclasts, respectively.

### 2.8. Immunofluorescent Analyses of Cells

For immunofluorescent staining, cells were first treated in Triton X-100 for half an hour before blocking with 10% BSA. Then, the cells were stained with primary antibodies against NFATc1 (Santa Cruz, CA, USA) or p65 (CST, MA, USA) at 4°C overnight. Finally, the samples were stained with secondary antibodies at room temperature for 1 h.

### 2.9. Actin Ring-Formation Assay

Briefly, the macrophage line cells were first stimulated with Rankl and varying doses of aucubin for 6 days. Then, the cultured osteoclasts were permeabilization with 0.5% Triton X-100 after being fixed with 4% paraformaldehyde. Subsequently, FITC-conjugated phalloidin was used to stain F-actin rings, and the nuclei were stained with 4′,6-diamidino-2-phenylindole (DAPI) dye. Finally, a scanning confocal microscopy (Nikon, Tokyo, Japan) was used to take fluorescence images. ImageJ software was used to analysis the number and size of F-actin rings.

### 2.10. Migration Assay

In the migration assay, MMECs were seeded on 6-well culture plates and cultured until the single cell layer was confluent. Then, cells were cultured in different treatments. MMECs were used in two parallel experiments. In the first experiment, MMECs were cultured in endothelial cell culture medium with or without aucubin. In the second experiment, MMECs were cultured in different conditioned medium (CM). According to the treatments, the groups were divided into the following groups: the vehicle (CM was harvested from RAW264.7 cells)+IgG (Abcam, Cambs, Britain) group, the Rankl (CM was gathered from RAW264.7 cells stimulated with Rankl)+IgG group, the Rankl+Aucubin (CM was gathered from RAW264.7 cells which were stimulated with Rankl and Aucubin at the same time)+IgG group, and the Rankl+Aucubin (CM collected from RAW264.7 cells stimulated with RANKL and aucubin)+PDGF-BB antibody (R&D, Minneapolis, USA) group. The cell monolayer was scratched with the tip of a pipette gun followed by washing with PBS. After 0, 24, and 48 hours, the wounds were acquired by microscopy and measured by ImageJ software.

### 2.11. Tube Formation Assay

To perform tube formation assays, 50 *μ*L/well of Matrigel (BD, USA) was spread on 96-well culture plates and incubated at cell incubator for half an hour. Then, MMECs were seeded on solidified gel in the 96-well plate and cultured under different treatments with a density of 1 × 10^4^ cells/well. MMECs were used in two parallel experiments similar to the scratch test. First, MMECs were cultured in endothelial cell culture medium with or without aucubin. Subsequently, MMEC groups were divided into the following groups: the vehicle (CM was harvested from RAW264.7 cells)+IgG group, the Rankl (CM was gathered from RAW264.7 cells stimulated with Rankl)+IgG group, the Rankl+Aucubin (CM was gathered from RAW264.7 cells which were stimulated with Rankl and Aucubin at the same time)+IgG group, and the Rankl+Aucubin (CM collected from RAW264.7 cells stimulated with RANKL and aucubin)+PDGF-BB antibody group. After 6 hours, tube formation was acquired by microscopy and measured by Image-Pro Plus 6 software.

### 2.12. Enzyme-Linked Immunosorbent Assay (ELISA)

The concentrations of CTX-1, OCN, PDGF-BB, and VEGF in the blood serum, bone tissue, or conditioned medium were measured using commercial ELISA Kits according to the manufacturers' instructions. The content of each group was detected by a microplate reader. PDGF-BB ELISA Kit was obtained from Cusabio (Wuhan, China). VEGF, CTX-1, and OCN ELISA Kit were purchased from Multi Sciences LTD (Hangzhou, China).

### 2.13. Real-Time RT-PCR

TRIzol reagent (Tiangen, Beijing, China), RevertAid™ First Strand cDNA Synthesis Kit (Thermo, Waltham, USA), and SuperReal PreMix Plus (Tiangen, Beijing, China) PCR Kit were used to perform the real-time RT-PCR analysis. First, total RNA was extracted by TRIzol reagent according to the protocols before reverse-transcribed into cDNA. Then, real-time RT-PCR was performed following the instructions. During the process, GAPDH was selected as the internal control, and the 2^−*ΔΔ*Ct^ method was used to evaluate relative gene expression. The specific primer sequences used for the experiments are as follows: GAPDH: 5′-AGTTCAACGGCACAGTCAAGG-3′, 5′-AGCACCAGCATCACCCCAT-3′; Atp6v0d2: 3′-AGCAAAGAAGACAGGGAG-5′, 5′-CAGCGTCAAACAAAGG-3′; NFATc1: 3′-CAACGCCCTGACCACCGATAG-5′, 5′-GGCTGCCTTCCGTCTCATAGT-3′; cathepsin K: 3′-CAGCAGAACGGAGGCATTGA-5′, 5′-CTTTGCCGTGGCGTTATACATACA-3′; PDGF-BB: 3′-CCTCGGCCTGTGACTAGAAG-5′, 5′-CCTTGTCATGGGTGTGCTTA-3′; DC-STAMP: 3′-GATCACCTGTGTTTTCCTATGC-5′, 5′-CAATCAAAGCGTTCCTACCTTC-3′; C-fos: 3′-CGGGTTTCAACGCCGACTA-5′, 5′-TTGGCACTAGAGACGGACAGA-3′; MMP-9: 3′-GCGTCGTGATCCCCACTTAC-5′, 5′-CAGGCCGAATAGGAGCGTC-3′; VEGF: 3′-GAGGTCAAGGCTTTTGAAGGC-5′, 5′-CTGTCCTGGTATTGAGGGTGG-3′; MMP-2: 3′-TCAAGTTCCCCGGCGATG-5′, 5′-AGTTGGCCACATCTGGGTTG-3′.

### 2.14. Western Blot Analysis

RIPA lysis buffer (Beyotime, Shanghai, China), 12% SDS-polyacrylamide gel electrophoresis (SDS-PAGE) and polyvinylidene fluoride (PVDF) membranes (Millipore, Billerica, MA, USA) were used to perform the Western blot analysis. Briefly, RIPA lysis buffer was used to obtain total proteins. Then, 25 *μ*g of protein was resolved by SDS-PAGE and subsequently transferred to PVDF membranes. Ten percent BSA in Tris-buffered saline mixed with Tween 20 (TBST) was used to block the PVDF membranes. The membranes were stained with primary antibodies against GAPDH (1 : 10000), PDGF-BB (1 : 1000), cathepsin K (1 : 1000), c-fos (1 : 1000), NFATc1 (1 : 1000), p-JNK (1 : 500), JNK (1 : 1000), p-p38 (1 : 1000), p38 (1 : 1000), p-p65 (1 : 1000), p65 (1 : 1000), p-IKB*α* (1 : 1000), or IKB*α* (1 : 1000) at 4°C for 8 h. Finally, the membranes were incubated with secondary antibodies (1 : 20,000, Rockland, USA) for 1 hour at room temperature. An Odyssey infrared imaging system was used to detect the integrated intensity for each group. Antibodies of NFATc1, PDGF-BB, and Emcn were purchased from Santa Cruz Biotechnology (Santa Cruz, CA, USA). p-JNK, JNK, p-p38, p38, p-IKB*α*, IKB*α*, p-p65, and p65 antibodies were obtained from Cell Signaling Technology (Danvers, MA, USA). c-Fos, CD31, OCN, and GAPDH antibodies were purchased from Abcam (Cambridge, MA, USA).

### 2.15. Statistical Analysis

The results in the present study are expressed as the mean ± standard deviation. The outcomes were from at least 3 independent replications. The Kolmogorov-Smirnov test was used to test the normality assumptions of experimental data. An independent samples *t*-test was used for the comparison between two groups, and one-way analysis of variance (ANOVA) followed by the Student Newman Keuls (S-N-K) post hoc analysis was used for the analysis among multiple groups. For the nonparametric data, the Kruskal-Wallis test was performed. All the data analysis was using SPSS 20.0 software, and statistical significance was measured by *P* < 0.05.

## 3. Results

### 3.1. Aucubin Inhibits OVX-Induced Bone Loss and Augments Type H Vessel Formation

**Figure 1:**
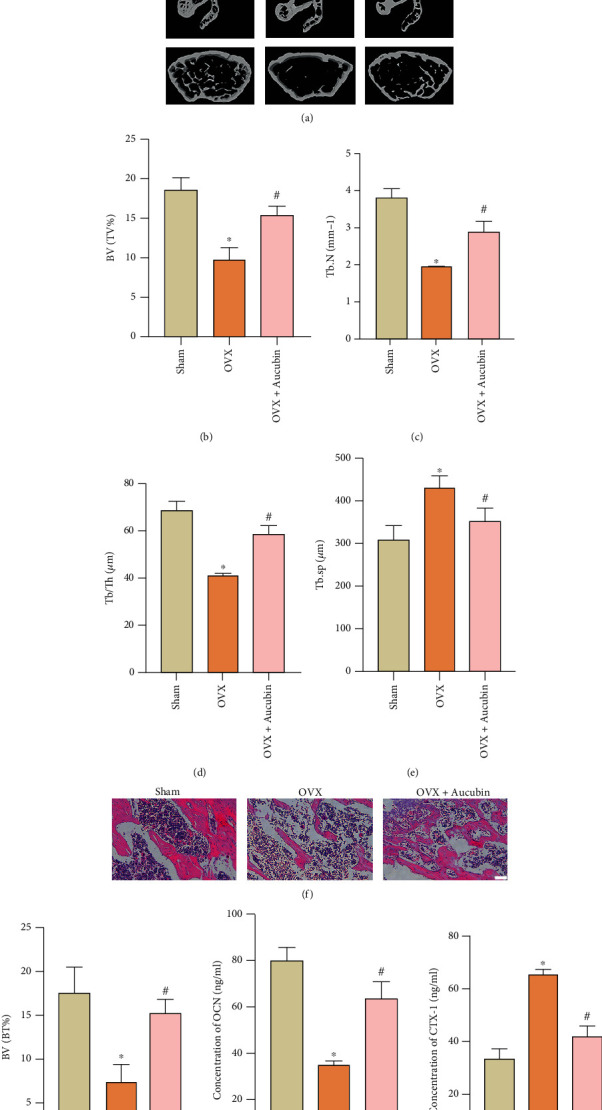
Aucubin ameliorated bone loss induced by OVX. (a) Micro-CT images and quantitative analysis. (b–e) of the mouse femurs from different groups. BV/TV: bone volume/total volume; Tb.N: trabecular number; Tb.Sp: trabecular separation; Tb.Th: trabecular thickness. (f) Representative haematoxylin and eosin (HE) staining of femurs; scale bar: 100 *μ*m. (g) Quantification of BV/TV% of HE staining. (h, i) The concentrations of OCN and CTX-1 in the serum were detected by ELISA. *n* = 5 per group. ^∗^*P* < 0.05 compared to the sham group. ^#^*P* < 0.05 compared to the OVX group.

In order to assess the effect of aucubin on bone loss and type H formation, an OVX mouse model was generated in the present study. OVX clearly impaired the bone structure compared to the sham group, while intraperitoneal injection of aucubin in OVX mice partly attenuated this bone loss as shown by micro-CT scans (Figure 1(a)). Correspondingly, quantitative analyses of the Tb.N, BV/BT%, and Tb.Th were significantly decreased after OVX surgery; however, these reductions were mitigated by the aucubin treatment (Figures 1(b)–1(d)). Aucubin also reduced the increased trabecular bone volume fraction (Tb.Sp) caused by OVX (Figure 1(e)). HE staining also verified that aucubin could rescue OVX-induced bone loss (Figures 1(f) and 1(g)). Consistent with this finding, the ELISA results indicated that aucubin significantly abolished the decrease in serum levels of OCN as well as the increase of CTX-1 content in serum induced by OVX (Figures 1(h) and 1(i)).

**Figure 2:**
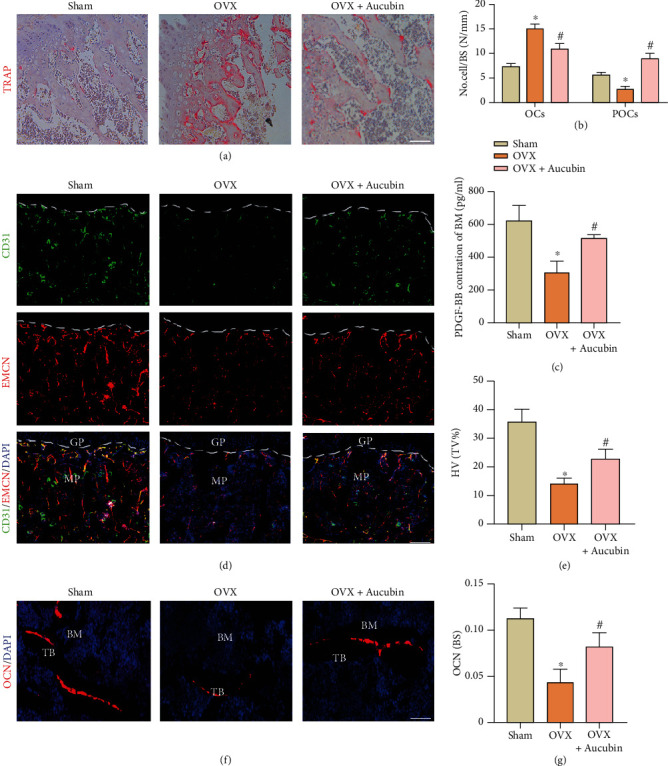
Aucubin represses osteoclast formation, promotes PDGF-BB secretion, and promotes type H vessel formation in OVX mice. (a) TRAP staining of the femora to calculate the numbers of osteoclasts and preosteoclasts of bone surface. Scale bar: 200 *μ*m. (b) Quantification of OCs and POCs per bone surface of different groups. BS: bone surface. (c) Quantification of PDGF-BB levels in bone marrow was detected by ELISA and analyzed. (d) Type H vessels were detected by CD31^hi^Emcn^hi^double IF. Scale bar: 100 *μ*m. (e) Type H vessels were quantitative analysis in each group. BM: bone marrow; GP: growth plate. *n* = 5 per group. (f) Representative images of IF staining of OCN, Scale bar: 50 *μ*m. (g) The quantification of OCN+cell surfaces/bone surfaces. TB: trabecular bone; BM: bone marrow. ^∗^*P* < 0.05 compared to the sham group; ^#^*P* < 0.05 compared to the OVX group.

TRAP staining was used to confirm the inhibitory function of aucubin on osteoclastogenesis in vivo. Consistent with the change in the bone resorption index in the serum, OVX mice had a larger number of multinucleated osteoclasts. However, aucubin administration significantly reduced the number of mature osteoclasts as well as increased the number of preosteoclasts (Figures 2(a) and 2(b)). The results confirmed that aucubin has an inhibitory role in osteoclast maturation. As the number of osteoclasts in each group was different, the content of PDGF-BB in bone marrow changed between groups. PDGF-BB decreased after the OVX operation. However, the PDGF-BB content increased after the aucubin intervention (Figure 2(c)). Emcn and CD31 double IF staining were used to confirm the effects of aucubin on angiogenesis of type H blood vessel. The quantity of type H vessels was decreased by OVX, and aucubin treatment results in a remarkable upregulation in this particular type vessel (Figures 2(d) and 2(e)). In addition, the influence of aucubin on bone formation was also detected. Aucubin partially alleviated the decrease in OCN in the bone surface caused by OVX (Figures 2(f) and 2(g)). The in vivo experiments suggested that aucubin treatment decreases the number of osteoclasts, promotes the angiogenesis of type H vessels, and increases bone formation. All animal experiments indicated that the preosteoclast-induced angiogenesis facilitation might be a potential mechanism by which aucubin protects skeleton.

### 3.2. Aucubin Increases the Preosteoclast Number and Inhibits Osteoclast Maturation

**Figure 3:**
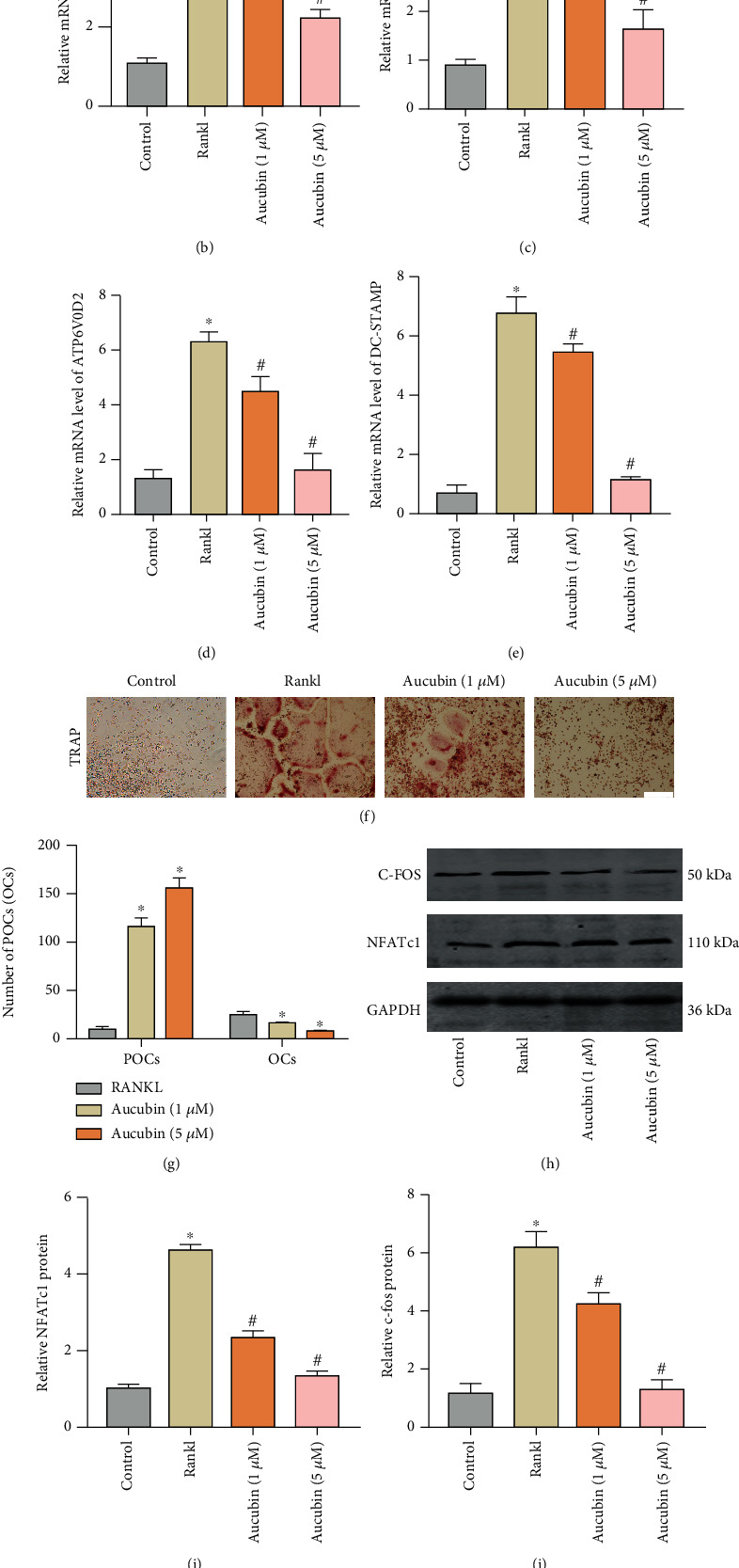
Aucubin increases the number of preosteoclasts in vitro. (a) Cell viability of RAW264.7 cells cultured with different concentrations of aucubin (0, 1 *μ*M, and 5 *μ*M) was analyzed via a CCK-8 assay. (b–e) Relative mRNA expression levels of NFATc1, c-FOS, DC-STAMP, and ATP6V0D2 were analyzed. (f) Representative images of TRAP staining on day 6. Scale bar: 200 *μ*m. (g) Quantification of osteoclasts (OCs) and preosteoclasts (POCs) on day 6. (h–j) Protein levels of c-Fos and NFATc1 were analyzed by WBs. (k) IF staining was performed to observe the location of NFATc1. Scale bar: 50 *μ*m. (l) The quantification of the relative NFATc1 fluorescence intensity in different groups. ^∗^*P* < 0.05 compared to the control group; ^#^*P* < 0.05 compared to the Rankl group.

CCK-8 assay was performed to detect the effect of aucubin on cell viability. The results were detected at 1, 2, and 3 days after administration of aucubin. There were no significant influences of aucubin on cell viability of RAW264.7 cells at low concentrations (1 *μ*M) or high concentrations (5 *μ*M) (Figure 3(a)). In order to detect the influence of aucubin on osteoclastogenesis, TRAP staining was performed 6 days after the RAW264.7 cells were stimulated by Rankl and administrated with or without aucubin. The results showed that the number of mature osteoclasts in RAW264.7 cells increased under Rankl induction while the fusion of TRAP+preosteoclasts was inhibited by the aucubin treatment, with the inhibitory effect more obvious with a high dose of aucubin (Figures 3(f) and 3(g)). These findings indicated that aucubin had the ability to prevent the maturation process of preosteoclasts into multinucleated osteoclasts. RT-PCR analysis was used to evaluate the expression of osteoclast-related mRNA and fusion-related mRNA, including NFATc1, cathepsin K (CTSK), ATP6V0D2, and DC-STAMP. As shown in Figures 3(b)–3(e), aucubin significantly suppressed osteoclast-related mRNA (NFATc1 and CTSK) as well as osteoclast fusion mRNA stimulated by RANKL in macrophage cells. In parallel tests, Western blot results indicated that c-Fos and NFATc1 protein were downregulated by the addition of aucubin (Figures 3(h)–3(j)). Next, IF staining was performed to assess the effect of aucubin on NFATc1, a key factor in osteoclast differentiation. The results showed the increase in NFATc1 induced by RANKL after 12 hours was partially offset by aucubin (Figures 3(k) and 3(l)). These parts of results indicate that aucubin depresses the fusion of preosteoclasts by inhibiting osteoclast-related factors.

### 3.3. Aucubin Promotes Angiogenesis via Increasing the Production of PDGF-BB In Vitro

**Figure 4:**
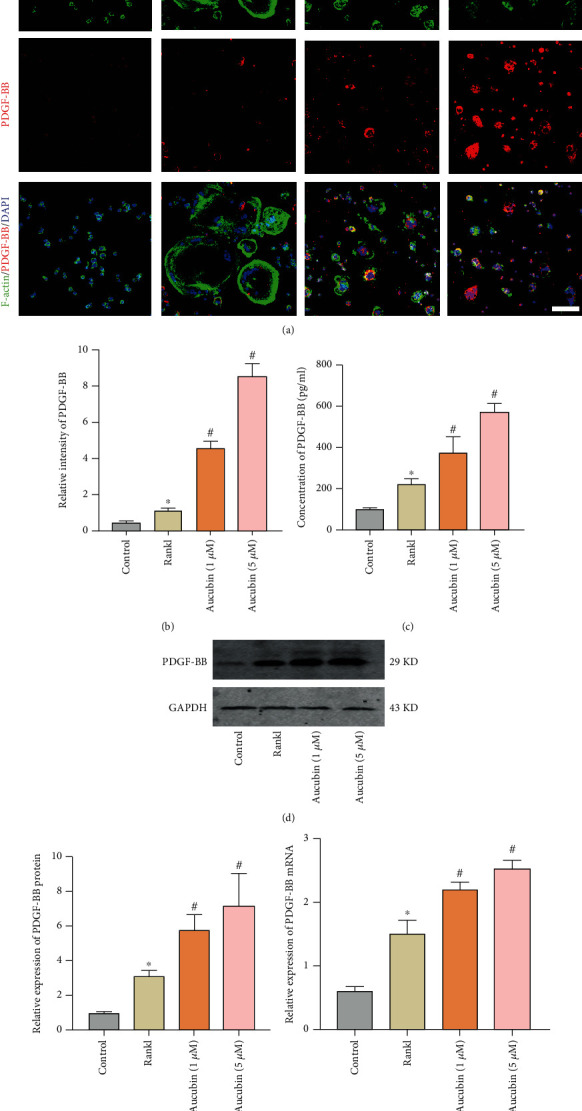
Aucubin augments the production of PDGF-BB in vitro. (a) IF staining was performed to observe the production of PDGF-BB. Scale bar: 50 *μ*m. (b) Quantification of the relative PDGF-BB fluorescence intensity. (c) Concentration of PDGF-BB in CM was analyzed by ELISA. (d) Protein levels of PDGF-BB were analyzed by WBs. (e) Quantification of PDGF-BB protein expression with different treatments. (f) Quantification of PDGF-BB mRNA expression with different treatments. ^∗^*P* < 0.05 compared to the control group; ^#^*P* < 0.05 compared to the Rankl group.

IF, ELISA, and Western blotting were performed to determine the influence of aucubin intervention on the production of PDGF-BB during the osteoclastic differentiation (Figures 4(a)–4(e)). The content of PDGF-BB increased by Rankl stimulation. Meanwhile, PDGF-BB further augmented with the inhibition of osteoclast precursor cell fusion by the aucubin treatment (Figures 4(a) and 4(b)). As confirmed by ELISA, Rankl increased the content of PDGF-BB in the supernatant and aucubin potentiated Rankl-induced PDGF-BB in a dose-dependent manner (Figure 4(c)). Consistent with the ELISA results, aucubin also upregulated the expression of PDGF-BB in protein and mRNA level (Figures 4(d)–4(f)). Thus, we suggested that aucubin promoted the secretion of PDGF-BB through raising the number of preosteoclasts.

**Figure 5:**
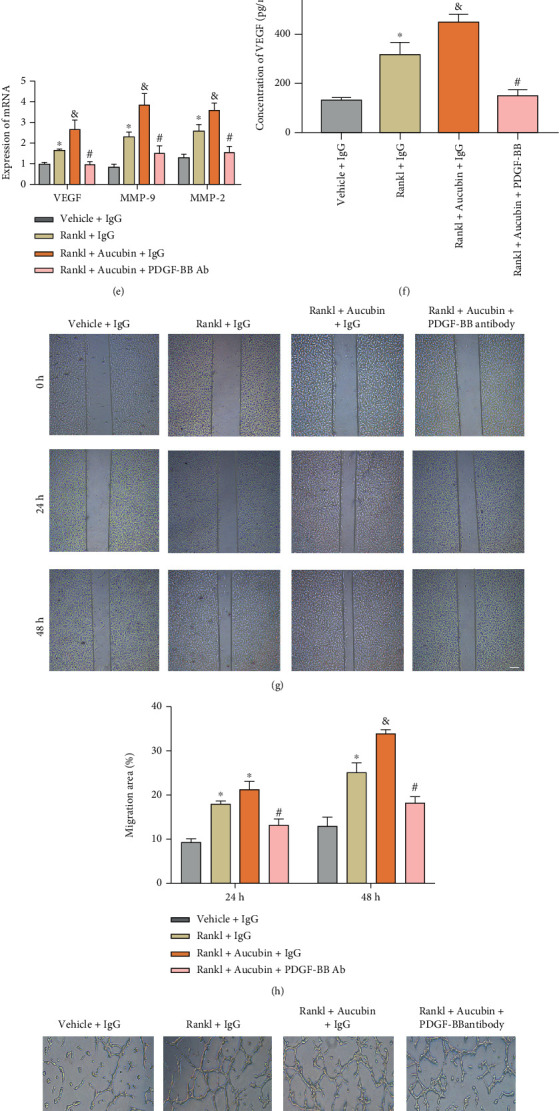
Aucubin promotes the proangiogenic effects of preosteoclasts on MMECs. (a) Representative images of the migration area of MMECs in different treatments. Scale bar: 200 *μ*m. (b) The quantification of the migration area of MMECs in different treatments. Scale bar: 200 *μ*m. (c) Representative images of tube formation in MMECs stimulated with different treatments. Scale bar: 200 *μ*m. (d) The quantification of tube formation in MMECs stimulated with different treatments. (e) Relative mRNA expression levels of VEGF, MMP-9, and MMP-2 in MMECs with different CMs. (f) Concentration of VEGF in CM was analyzed by ELISA. (g) Representative images of the migration area of MMECs in different treatments. Scale bar: 200 *μ*m. (h) The quantification of the migration area of MMECs in different treatments. ^∗^*P* < 0.05 compared to the vehicle+IgG group; ^&^*P* < 0.05 compared to the Rankl+IgG group; ^#^*P* < 0.05 compared to the Rankl+Aucubin+IgG group. (i) Representative images of tube formation in MMECs stimulated with different treatments. Scale bar: 200 *μ*m. (h) The quantification of tube formation in MMECs stimulated with different treatments. ^∗^*P* < 0.05 compared to the vehicle+IgG group; ^&^*P* < 0.05 compared to the Rankl+IgG group; ^#^*P* < 0.05 compared to the Rankl+Aucubin+IgG group.

To assess whether aucubin had the direct effect on angiogenesis, the scratch wound assay (Figures 5(a) and 5(b)) and tube formation assay (Figures 5(c) and 5(d)) were performed. The quantitative measurements revealed that aucubin had no significant effect on the migration ability and tube formation ability of MMECs. In order to confirm the effect of aucubin in preosteoclast-stimulated angiogenesis and migration, MMECs were cultured in different groups: the vehicle+IgG group, the Rankl+IgG group, the Rankl+Aucubin+IgG grpup, and Rankl+Aucubin+PDGF-BB antibody group. As shown in the scratch test, the migration ability of MMECs was significantly enhanced in the Rankl+IgG group compared to the vehicle+IgG group, and the supernatant of the aucubin+Rankl group enhanced the effect when combined with that of the Rankl group (Figures 5(g) and 5(h)). To further verify the changes mediated by PDGF-BB, the PDGF-BB-neutralizing antibody was further added to the RANKL+aucubin group, and the cell migration was inhibited. The results of the tube formation experiment are similar to those of the scratch test. The CM of Aucubin further expanded the total tube length compared to the Rankl+IgG group. However, this increase was attenuated by PDGF-BB-neutralizing antibodies (Figures 5(i) and 5(j)). As evidenced by PCR, the expression of MMP-9, MMP-2, and VEGF mRNA of the aucubin+Rankl+IgG group was increased when compared to that of the Rankl+IgG group and PDGF-BB-neutralizing antibodies eliminated the increase of MMP-9, MMP-2, and VEGF mRNA caused by aucubin CM (Figure 5(e)). Consistent with the PCR results, the increased concentration of VEGF in the aucubin+Rankl+IgG group was blocked by neutralizing antibodies against PDGF-BB (Figure 5(f)). In summary, aucubin promotes angiogenesis via increasing the production of PDGF-BB of preosteoclasts.

### 3.4. Aucubin Inhibits RANKL-Induced MAPK and NF-*κ*B Signaling

**Figure 6:**
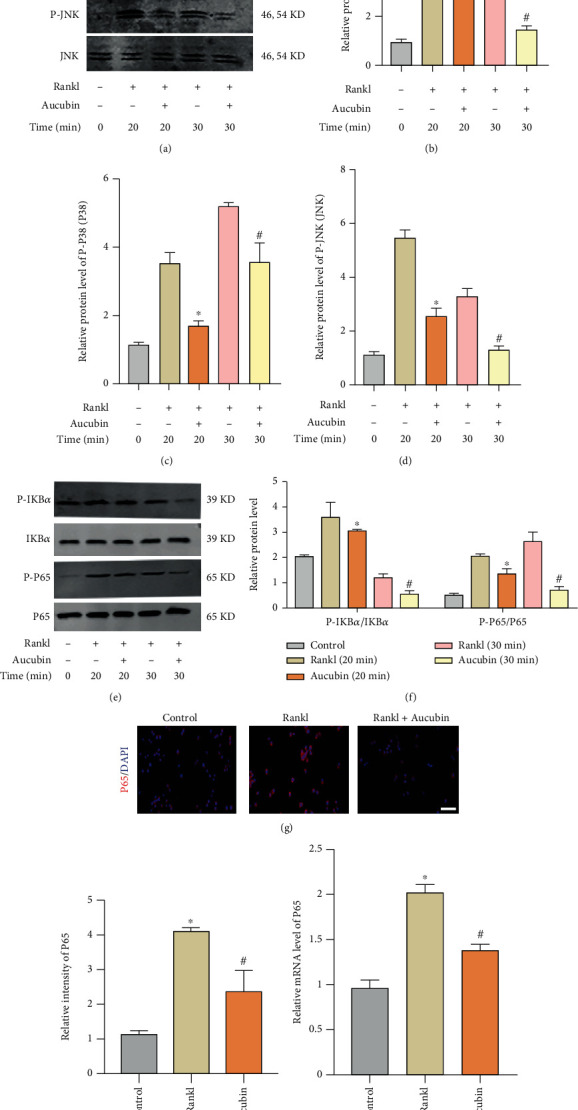
Aucubin inhibits the MAPK and NF-*κ*B signaling pathways. (a) Protein levels of the MAPK family were analyzed by WBs. (b–d) Quantification of MAPK family protein expression. ^∗^*P* < 0.05 compared to the Rankl group at 20 min; ^#^*P* < 0.05 compared to the Rankl group at 30 min. (e) Protein levels of the NF-*κ*B family were analyzed by WBs. (f) Quantification of NF-*κ*B family protein level. ^∗^*P* < 0.05 compared to the Rankl group at 20 min; ^#^*P* < 0.05 compared to the Rankl group at 30 min. (g) Location of p65 fluorescence was observed via IF. Scale bar: 50 *μ*m. (h) Relative p65 fluorescence intensity was analyzed. ^∗^*P* < 0.05 compared to the control group; ^#^*P* < 0.05 compared to the Rankl group. (i) Relative p65 mRNA was analyzed. ^∗^*P* < 0.05 compared to the control group; ^#^*P* < 0.05 compared to the Rankl group.

In order to elucidate the mechanism of aucubin on osteoclast formation and PDGF-BB production, the MAPK and NF-*κ*B signaling pathways, which play important roles in osteoclastogenesis, were detected. Western blots confirmed that aucubin significantly suppressed the MAPK signaling pathway. The active forms of ERK, p38 and JNK at 20 and 30 minutes was inhibited by aucubin treatment compared with that of the Rankl group (Figures 6(a)–6(d)). To further clarify whether NF-*κ*B signaling took part in the mechanisms underlying the effect of aucubin, IKB*α* and p65 protein were checked (Figures 6(e) and 6(f)). The outcomes revealed that aucubin suppressed the activation of IKB*α* (Figures 6(e) and 6(f)). IKB*α* regulates the activity of p65 in the NF-*κ*B signaling pathway. In addition, the phosphorylation of p65 was also inhibited by aucubin (Figures 6(e) and 6(f)). Results of IF staining and qRT-PCR further showed that NF-*κ*B translocation to the nucleus was activated by Rankl and inhibited by aucubin (Figures 6(g) and 6(i)). The above results indicated that aucubin inhibited Rankl-induced osteoclastogenesis through the MAPK and NF-*κ*B signaling pathways.

## 4. Discussion

Osteoporosis, characterized by quantitative and qualitative deterioration of bone, has become one of the most serious chronic diseases [21]. The imbalance of bone formation and bone resorption is the most direct cause of osteoporosis. Recent studies have confirmed that bone microenvironment, especially the vascular system, plays a crucial part in maintaining the normal progression of bone metabolism [22]. Type H vessels that are mostly regulated by PDGF-BB are vital to bone remodeling [4]. Moreover, previous studies have affirmed that the suppression of osteoclasts differentiation at the precursor stage could promote the production of PDGF-BB [23–25]. Thus, blocking bone absorption and enhancing osteogenesis by promoting type H vessels might represent a new direction for the treatment of bone-lost diseases. In this research, it is demonstrated that aucubin inhibited the preosteoclast fusion into multinucleated osteoclasts, promoted the content of PDGF-BB, increased the quantity of type H vessels, and eventually takes precautions against OVX-induced bone loss in vivo.

Aucubin is derived from *Eucommia ulmoides*, a traditional Chinese medicine that has bone protection effects. As an iridoid glycoside compound, aucubin displays anti-inflammatory and antioxidative effects [26]. A recent study also demonstrated that aucubin regulates neovascularization in hindlimb ischaemia [19]. However, its effect on the bone is not as well understood. In the present experiments, we built an OVX model to detect the effect of aucubin on bone metabolism. Intraperitoneal injection of aucubin can significantly increase bone mass caused by OVX, which agreed with the outcomes reported by Li et al. [17, 18]. Meanwhile, the contents of PDGF-BB in bone marrow increased after aucubin intervention, suggesting that aucubin might increase bone mass through the enhancing angiogenesis induced by PDGF-BB. Further immunofluorescence results were consistent with these findings and showed that aucubin could upregulate the quantity of type H blood vessels characterized by CD31 and endomucin. Despite the low quantity, type H vessels undertake the process of bone formation by closely surrounded by osteoprogenitors. In addition to providing blood supply, type H vessels can secrete many factors that stimulate the proliferation and osteogenesis of osteoprogenitors. Bone loss related to ageing and bone diseases is at least partly resulting from the changes in quantity and function of type H vessels. Conform with previous researches, the present study also shows that the content of type H vessels is related to OVX-induced bone loss. Researches have shown that type H vessels play a vital role in the treatment of osteoporosis, fracture, and other bone-loss disease models [27, 28]. In the present study, aucubin attenuated the decreased OCN in serum and bone tissue caused by OVX and enlarged the number of type H vessels. This result shows that aucubin might be a candidate for osteoporotic treatment.

In the present study, aucubin alone did not increase angiogenesis in vitro indicating that the augmented angiogenesis caused by aucubin might due to an indirect effect. Furthermore, we found an upregulation of PDGF-BB in the CM after the administration of aucubin. And the remarkably enhanced angiogenic activities of MMECs were accompanied by the increase of PDGF-BB level in the cultured CM. Meanwhile, VEGF, the important marker of angiogenesis, was upregulated after the administration of aucubin+Rankl CM. Previous studies had shown that the levels of PDGF-BB and VEGF were strongly correlated [29]. PDGF is an important upstream mediator in hypoxia-induced VEGF up-regulation [30]. And PDGF-BB has been reported to induce the secretion of VEGF in a manner dependent on both Akt and MAPK activation in ovarian cancer [31]. The enhanced angiogenic activities induced by aucubin+Rankl CM were abolished by the intervention of PDGF-BB-neutralizing antibodies, confirming that the upregulation of angiogenic activities was induced by PDGF-BB. Previous studies have confirmed that PDGF-BB could enhance the type H vessels angiogenesis and subsequent osteogenesis during bone remodeling [32]. PDGF-BB is an important member of the PDGF family which is important in the process of the proliferation, migration, and differentiation of endothelial progenitor cells. It exerts its function by binding to its specific receptor PDGF receptor *β* (PDGFR*β*) mitogen-activated kinase and inducing a signaling cascade [33]. Recently, preosteoclasts have been confirmed as the most important source of PDGF-BB. In the present experiment, aucubin administration led to an accumulation of PDGF-BB in the CM as it increased the number of preosteoclasts. Further mechanism experiments showed aucubin increased the number of preosteoclasts by decreasing the expression of NFATc1, DC-STAMP, and ATPV0D2 mRNA induced by Rankl. NFATc1 is a vital regulator during the process of osteoclastogenesis. In addition to controlling the expression of the osteoclast differentiation related genes TRAP and cathepsin K, NFATc1 also participates in the multinucleation of osteoclasts through cell fusion molecules [34, 35]. DC-STAMP and ATPV0D2 participate in the process of cell-cell fusion [36, 37]. The fusion of macrophage cells into foreign body giant cells was completely abrogated in DC-STAMP-deficient mice [38]. In addition, knockdown of CTSK has been reported to inhibit the maturation of osteoclasts [10]. The decreased relative expression of osteoclast marker genes, including CTSK, DC-STAMP, ATP6V0D2, c-Fos, and NFATc1, demonstrated an inhibitory effect of aucubin on the differentiation of preosteoclasts into osteoclasts. The above results suggest that aucubin promotes type H vessel angiogenesis by inhibiting osteoclast fusion to produce more PDGF-BB.

After confirming that aucubin inhibited osteoclast fusion to promote type H vessel angiogenesis, we then explored the mechanism by which aucubin suppress preosteoclast fusion. In this research, we found that aucubin inhibited the activation of the MAPK signaling pathway. MAPK and NF-*κ*B signaling pathways are vital during Rankl-induced osteoclast differentiation [39]. After Rankl administration, the activation of the MAPK family, namely, p38, JNK, and ERK, increased. The ERK signaling is involved in the survival, proliferation, and differentiation of osteoclasts [40]. Bone marrow-derived macrophages isolated which were lacking of JNK1 showed reduced osteoclast differentiation activity [41]. Activated p38 directly stimulates NFATc1 to enhance the differentiation of osteoclasts [42]. Meanwhile, aucubin was found to inhibit the phosphorylation and degradation of IKB*α* in the present study, which is an important part of NF-*κ*B signaling pathway and suppresses p65 nuclear translocation by binding to p65. The IF staining results also indicated that aucubin inhibited the Rankl-induced activity of p65 signaling pathway. Recently, the PDGF-B promoter region was confirmed to contain an NF-*κ*B binding domain [43]. Thus, the decrease in p65 activation into the nucleus directly inhibited the transcription of PDGF-BB. Taken together, we conclude that aucubin might enhance preosteoclast PDGF-BB-induced angiogenesis by inhibiting MAPK/NF-*κ*B signaling and ultimately accelerate osteogenesis and prevent bone loss induced by OVX.

## 5. Conclusions

In conclusion, our research demonstrated that aucubin has the ability to inhibit multinucleated osteoclast maturation and promote the formation of type H vessels in OVX mice. The underlying mechanism may be that aucubin increases preosteoclast and subsequent PDGF-BB-induced angiogenesis by inhibiting MAPK/NF-*κ*B signaling. All the findings indicate that aucubin might be an anti-bone-loss drug candidate which needs further research.

## Data Availability

The data used to support the findings of this study are available from the corresponding author upon request.
